# Gonadal Transcriptome Analysis Reveals the lncRNA–mRNA Pair in Sea Cucumber *Holothuria leucospilota*

**DOI:** 10.3390/genes16111293

**Published:** 2025-10-30

**Authors:** Jing Zhang, Jingwei Yu, Yang Zhang, Meiyao Su

**Affiliations:** 1 School of Life Sciences and Biopharmaceutics, Guangdong Pharmaceutical University, Guangzhou 510006, Chinaasakawa080777@gmail.com (M.S.); 2 Guangdong Provincial Key Laboratory of Applied Marine Biology, Guangzhou 510301, China

**Keywords:** lncRNA, sea cucumber, *Holothuria leucospilota*, transcriptome

## Abstract

**Background/Objectives:** Long non-coding RNA (lncRNA) was structurally similar to mRNAs, yet they could not be translated into proteins. While an increasing number of reports have systematically identified and described lncRNA in model species, information about non-model species remains scarce. Sea cucumber *Holothuria leucospilota* could be used for both medicinal and food purposes, which have high economic value, gradually attracting the attention of researchers. **Methods:** In this research, we constructed lncRNA library and compared the difference in lncRNA expression profiles between testis and ovary of sea cucumber *H. leucospilota*. To elucidate the molecular interactions between lncRNA and mRNA, we computationally predicted potential complementary binding sites through analysis of both cis- and trans-acting antisense mechanisms. Subsequent Gene Ontology (GO) and Kyoto Encyclopedia of Genes and Genomes (KEGG) pathway analyses demonstrated that the identified target genes are potentially involved in the regulatory pathways governing gonad development. **Results:** Quantitative reverse transcription PCR analysis showed that MSTRG.32831.1-*sox9* and MSTRG.57315.1-*mthfr* exhibited a high expression pattern in testis; while MSTRG.11041.1-*mafa* and MSTRG.11074.1-*macf1* showed a high expression pattern in the ovary. **Conclusions:** Deciphering lncRNA–mRNA expression patterns may uncover fundamental principles governing reproductive regulation in marine invertebrates. This discovery not only deepens understanding in this field but also provides valuable comparative insights for developmental biology.

## 1. Introduction

High-throughput sequencing demonstrated that merely 2% of eukaryotic genomic sequences encode proteins, whereas 98% constitute non-coding regions. This reveals extensive transcription of non-coding RNAs (ncRNAs) [1]. Among these, long non-coding RNAs (lncRNAs) are defined as transcripts exceeding 200 nucleotides, synthesized by RNA polymerase II/III and lacking protein-coding capacity [2]. Indeed, classical long non-coding RNAs (lncRNAs) including X-inactive specific transcript (XIST) were identified decades ago [3]. However, due to their relatively low expression levels compared to protein-coding transcripts, lncRNAs were frequently overlooked by researchers in early studies. Recent advances have demonstrated that lncRNAs play crucial regulatory roles in various aspects of gonad development, encompassing testicular development [4], spermatogenesis [5], ovarian development [6], and oogenesis [7].

Regulatory function of lncRNA in echinoderms has gradually attracted the attention of researchers. Full-length transcriptome sequencing of purple sea urchin (*Heliocidaris crassispina)* generated 5098 long non-coding RNAs [8]. Chromosome-level genome of black sea urchin (*Arbacia lixula*) assembly characterized 48,596 non-coding RNAs, including lncRNA [9]. Currently, the study of lncRNA in sea cucumber is mainly focused on *Apostichopus japonicus*. Novel lncRNA001074 participates in the low salinity-induced response in the sea cucumber *A. japonicus* by targeting the let-7/NKAαxis [10]. LncRNA-microRNA-Gene network was constructed in *H. leucospilota* and *Holothuria glaberrima* during LPS challenge and radial organ complex regeneration [11]. Host lncRNAs in response to *Vibro splendidus* infection was characterized as efficient miRNA sponges in sea cucumber [1]. Transcriptome analysis characterized the 14,711 lncRNAs in *A. japonicus* [12]. Differentially expressed lncRNAs were identified as responses to environmental stresses in *A. japonicus.* High-throughput analyses demonstrated that environmental stressors significantly modulated the expression profiles of multiple lncRNAs in *A. japonicus*, thereby exerting regulatory influence on critical biological processes such as immune responses, energy metabolism, and cell cycle dynamics [13]. Furthermore, research revealed that m6A-dependent post-transcriptional regulation of lncRNAs was predominantly mediated by RNA methylation modifications in sea cucumbers exhibiting varying severity stages of skin ulceration syndrome—a prevalent pathology characterized by progressive dermal lesions and systemic metabolic disruption [14].

*H. leucospilota* represents a commercially significant marine species in aquaculture due to its high nutritional value. Over recent decades, increasing market demand for sea cucumber products has driven substantial expansion within the aquaculture industry. Understanding the regulation of gonadal tissue development could help solve the efficiency problem of artificial breeding of sea cucumber. It has been reported that lncRNA participated in regulating gonad development of marine invertebrate biology [15]. In our research, Illumina sequencing and De novo assembly of long non-coding RNAs in gonadal were detected in sea cucumber *H. leucospilota.* A total of 212 differentially expressed lncRNAs (DELs) were identified between ovary and testis. In the result of qPCR, MSTRG.32831.1-*sox9* and MSTRG.57315.1-*mthfr* showed testis preference expression pattern; however, the ovary expression of MSTRG.11041.1-*mafa* and MSTRG.11074.1-*macf1* were significantly higher than testis. The construction of a long non-coding RNA library of sea cucumber *H. leucospilota* supplemented the small RNA database of echinoderms. Our research laid the foundation for exploring the functions of sea cucumber long non-coding RNAs.

## 2. Materials and Methods

### 2.1. Specimen Acquisition

The experimental specimens of *H. leucospilota* were procured from coastal waters near Maoming, Guangdong Province, China. Mature individuals at gonadal developmental stage III were selectively sampled, meeting the following criteria: gonad mass ranging 30–50 g with oocyte diameter exceeding 150 μm [16]. Morphometric analysis revealed sexual dimorphism: males averaged 200 ± 2 mm in body length (SD = 3 mm) and 205 ± 3 g in wet weight, while females measured 203 ± 2 mm (SD = 3 mm) with an estimated weight of 207 ± 3 g (SD = 2 g). Following low-temperature transit to laboratory facilities, specimens were maintained in recirculating aquaculture systems under controlled conditions: salinity 32%, pH 8.0 ± 0.1, and constant temperature regime of 25 ± 1 °C. During the 3-day acclimatization period preceding experimentation, animals were fed ad libitum with Spirulina powder. Sex determination was performed via gross morphological examination, with females exhibiting characteristic red gonadal tissue contrasting with the white coloration in males. All procedures strictly adhered to the ARRIVE (Animal Research: Reporting of In Vivo Experiments) Guidelines 2.0 for ethical animal research [17] and the National Institutes of Health Guide for the Care and Use of Laboratory Animals (NIH Publications No. 8023, revised 1978). The dissected gonads were frozen and stored in liquid nitrogen. The field studies did not involve endangered or protected species.

### 2.2. Small RNA Sequencing Workflow: From Total RNA Extraction to Library Preparation

Gonadal total RNA was purified utilizing the RNeasy Mini Kit (Qiagen, Redwood City, CA, USA) following the manufacturer’s standardized protocols. Subsequent procedures involved the construction of small RNA sequencing libraries through adapter ligation and reverse transcription, followed by Illumina-based next-generation sequencing to generate comprehensive small RNA profiles. The quality and concentration of RNA were measured using NanoDrop 1000 spectrophotometer (Thermo, Waltham, MA, USA). Total RNA was extracted from gonadal tissue samples of three female and three male individuals in biological replicates. Each sea cucumber individually underwent independent library preparation and sequencing. These RNA samples were subsequently subjected to Solexa sequencing analysis. For small RNA library preparation, RNA fragments ranging from 16 to 30 nucleotides were size-selected using 15% denaturing polyacrylamide gel electrophoresis (PAGE). Following size selection, adapters were enzymatically ligated to both 5′ and 3′ ends of the small RNAs. These adapter-modified molecules served as templates for cDNA synthesis through reverse transcription, followed by PCR amplification and ultimately high-throughput sequencing on the Illumina HiSeq 2000 platform.

Raw reads underwent quality control using fastp to generate clean reads, whose Q20, Q30, and GC content metrics were computed for downstream analysis [18]. Clean reads were mapped to the *H. leucospilota* genome database (GenBank assembly: GCA_029531755.1) via Bowtie2 [19]. Alignment files were processed with StringTie for transcript assembly [20]. Transcript expression levels were estimated via RNA-Seq by Expectation-Maximization (RSEM), followed by isoform abundance quantification (FPKM) using Cufflinks [21]. Normalized data were analyzed for differential expression in DESeq, with significance thresholds set at adjusted *p* ≤ 0.1 and |log_2_FC| ≥ 1 for coding genes and lncRNAs [22]. This workflow was also applicable to library preparation and sequencing analysis of long transcripts.

### 2.3. Identification of lncRNA

The initial sequencing image was converted into raw nucleotide sequence data via base calling algorithms. Subsequently, quality control procedures were performed to generate clean data by eliminating the following: (1) low-quality reads (defined as sequences containing > 4 bases with quality scores < 13), (2) reads contaminated with 5′ primer sequences, (3) reads lacking 3′ adapters, (4) reads without insert fragments, (5) reads shorter than 18 nucleotides, and (6) poly(A)-containing reads. For RNA classification, non-target RNAs including miRNAs, rRNAs, tRNAs, snRNAs, and snoRNAs were filtered out through alignment against reference databases (GenBank and Rfam). Transcripts were initially classified as protein-coding or non-coding based on existing annotations. The identification of long non-coding RNAs (lncRNAs) was performed through an integrated computational pipeline applying multiple criteria: (1) transcript length > 200 bp, (2) single-exonic structure, and (3) negative coding potential predictions from five independent algorithms: HMMER (for protein domain analysis against Pfam database) [23], Pfam-scan [24], Coding Potential Calculator (evaluating ORF features and sequence conservation via BLASTX tool (https://blast.ncbi.nlm.nih.gov/Blast.cgi, accessed on 5 October 2025) against Nr database with E-value cutoff 1 × 10^−3^) [25], CNCI [26], and phyloCSF v0.6(assessing evolutionary signatures of protein-coding potential) [27]. CNCI could efficaciously differentiate coding and non-coding sequences independent of assigned annotations. PhyloCSF was employed to assess a muti-species nucleotide sequence alignment in order to estimate whether it corresponds to a conserved coding region by calculating the replacement frequency of phylogenetic codon [27]. Long non-coding RNA (lncRNA) transcripts were assembled using StringTie v2.2.4 [28], and their expression abundance and variation were quantified as Fragments Per Kilobase of exon model per Million mapped fragments (FPKM) using RSEM software v1.3.4 [29].

### 2.4. Predicting Target Genes for lncRNAs

Guided by lncRNA functional principles, we predicted lncRNA target genes employing antisense and cis-/trans-acting regulatory theory. These predictions were filtered using existing protein-coding messenger RNA datasets. Following cis-/trans-acting regulatory principles, we categorized the lncRNA–mRNA pairs: cis-acting targets were those within ~10 kb upstream or downstream of the lncRNA, whereas trans-acting targets were selected based on significant expression correlation (Pearson’s |r| ≥ 0.95 and *p*-value < 0.05).

### 2.5. Validation of lncRNA Expression Levels by Quantitative Real-Time PCR

We validated the results using real-time quantitative PCR (qPCR) experiments performed on gonadal tissue samples from different individuals within the same collection batch and adhering to the same standard. The RNA extraction method was consistent with the method described in 2.2. Total RNA (1 μg) underwent reverse transcription with the PrimeScript™ One Step RT-PCR Kit Ver.2 (Takara, Tokyo, Japan) following the manufacturer’s protocol, involving a 60 min incubation at 37 °C followed by 5 s enzyme inactivation at 85 °C. Quantitative real-time PCR amplification was performed using Platinum SYBR Green qPCR SuperMix-UDG (Invitrogen, Carlsbad, CA, USA) with lncRNA-specific primers (Supplementary Table S1), using β-actin as the endogenous control [30]. Relative lncRNA expression levels were calculated via the 2^−ΔΔCT^ method. Triplicate experimental data are presented as mean ± standard error (SE), with statistical significance determined at *p* < 0.05 through SPSS18 analysis.

### 2.6. Statistical Analysis

An unsupervised principal component analysis (PCA) was conducted utilizing the prcomp statistical function in R (version 4.2.2), with input data preprocessed by unit variance scaling [31]. Subsequent correlation analyses included the following: (1) Pearson correlation coefficients to evaluate relationships both among differentially expressed genes (DEGs) and between RT-PCR and RNA-seq expression profiles [32]; and (2) non-parametric Spearman correlation for additional DEG interdependency assessment [33]. All computational analyses were performed in the R environment (www.r-project.org) [34], while experimental data visualization and processing were accomplished using GraphPad Prism (version 9.0) [35].

## 3. Results

### 3.1. Assembling the Gonadal Long Non-Coding RNAs Through Illumina Sequencing

The sequencing output comprised 64.05 GB of raw data containing 426,936,528 paired-end reads, with 216,119,028 reads originating from ovarian tissue and 210,817,500 from testicular samples. Following stringent quality control procedures involving adapter trimming and low-quality read removal, high-fidelity datasets were generated consisting of 215,438,907 ovarian clean reads (retention rate: 99.68%) and 210,102,058 testicular clean reads (retention rate: 99.66%). After assembling the filtered sequencing reads, we generated 23,603 transcripts with a minimum length of 200 base pairs. After optimization, 17,491 unigenes were generated (Table S2). All raw reads were submitted into the NCBI Sequence Read Archive database with accession numbers PRJNA1198409.

**Figure 1:**
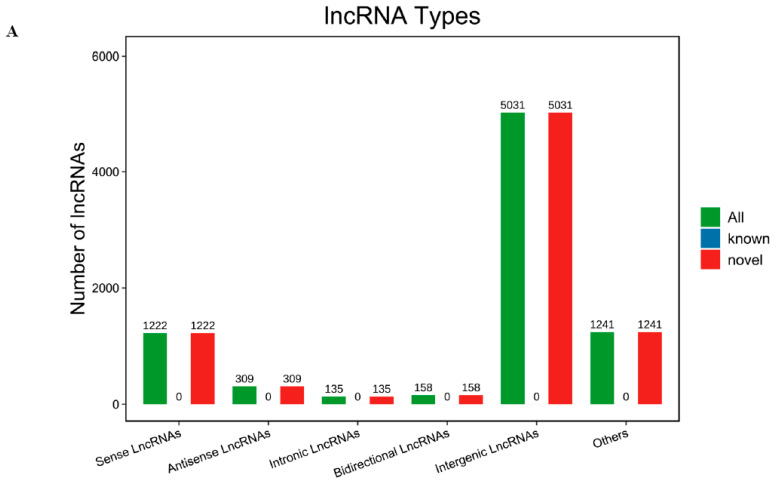

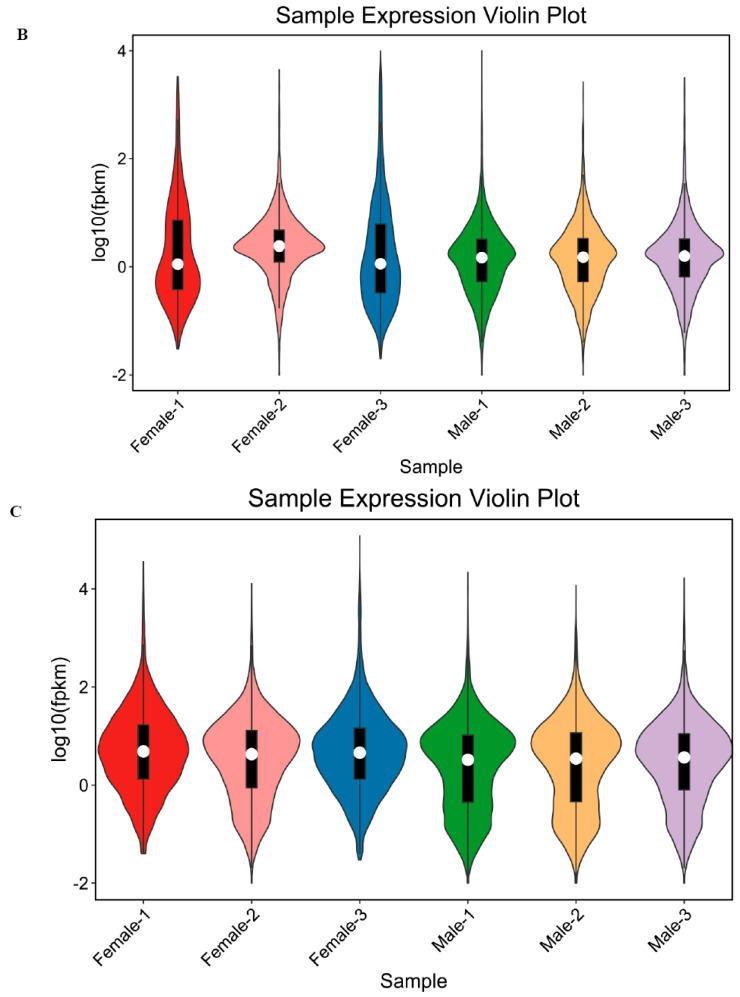
lncRNA types of gonadal (**A**); FPKM analysis of lncRNA in gonadal (**B**); FPKM analysis of mRNA in gonadal (**C**).

Through RNA-Seq analysis of ribodepleted mature gonadal RNA libraries, 8096 previously uncharacterized long non-coding RNAs were discovered based on their structural annotation criteria, with none matching existing lncRNA databases. The 8096 novel lncRNAs were categorized by their genomic localization relative to coding genes, comprising the following: intergenic (5031), intronic (135), sense (1222), antisense (309), bidirectional (158), and others (1241) (Figure 1A). Intergenic lncRNAs were the most numerous, accounting for 62.14% in total. Intronic lncRNAs were the least abundant, accounting for only 1.66% in the summary (Table S3).

Using the Fragments Per Kilobase of exon model per Million mapped fragments (FPKM) method to analyze lncRNA (Figure 1B) and mRNA (Figure 1C) expression levels in ovary and testis tissue of *H. leucospilota*, it was found that the expression patterns of lncRNAs were similar to mRNAs, but with some differences. Compared with lncRNAs, most mRNAs had relatively high expression levels.

### 3.2. LncRNA Differential Expression Pattern Between Ovary and Testis of H. leucospilota

**Figure 2:**
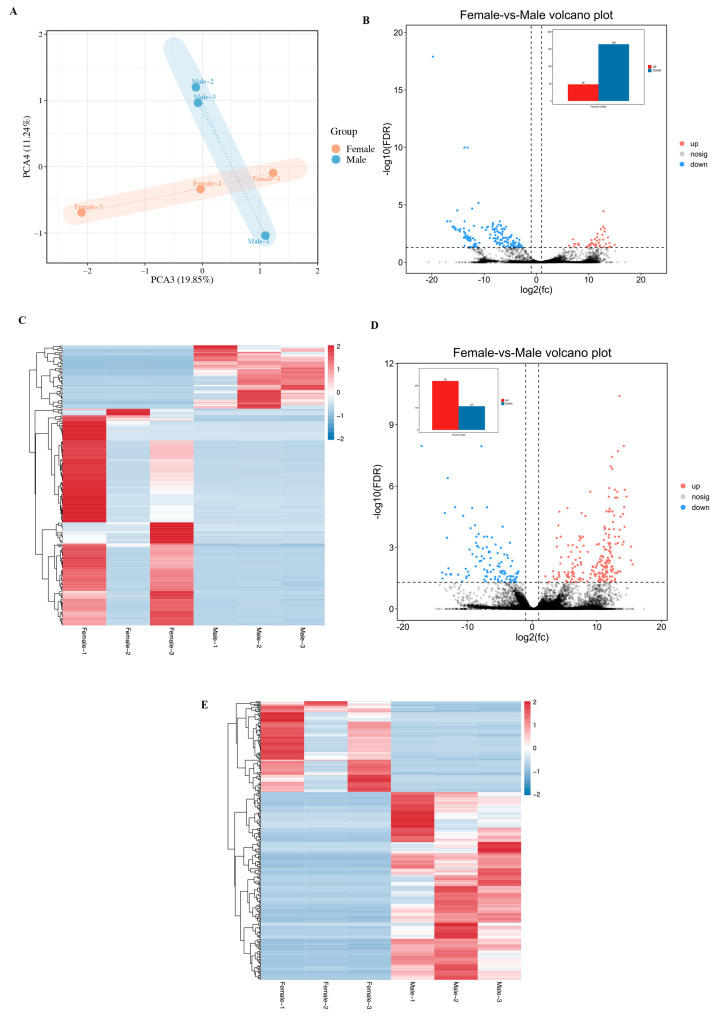
PCA of gonadal samples in *H. leucospilota* (**A**); Differentially expressed gonadal lncRNAs (**B**); heatmap of differentially expressed lncRNAs between ovary and testis samples (**C**); DEGs in gonadal of *H. leucospilota* (**D**); and heatmap of DEGs (**E**).

Based on the lncRNA expression levels of each sample, we used principal component analysis (PCA) to understand the repeatability between ovary and testis. As shown in Figure 2A, ovary and testis tissues were clearly separated, suggesting significant differences between the two tissues. Samples within the gonadal tissue group tend to cluster together, indicating positive consistency. A total of 212 differentially expressed lncRNAs (DELs) were identified in the gonadal tissues between female and male individuals (Table S4). Compared with female samples, 48 lncRNAs were upregulated and 164 lncRNAs were downregulated in male samples (Figure 2B). Figure 2C showed the heat map of unique expression profiles of long non-coding RNAs in ovarian and testis samples of *H. leucospilota.*

Totally, 328 differentially expressed mRNAs were detected in the samples (Table S5). Differential expression analysis revealed 212 upregulated and 107 downregulated mRNAs in male versus female samples (Figure 2D). The unique expression signatures of mRNAs in *H. leucospilota* ovary and testis were visualized in Figure 2E.

### 3.3. Association Analysis Between lncRNA and mRNA in Gonadal of H. leucospilota

**Figure 3:**
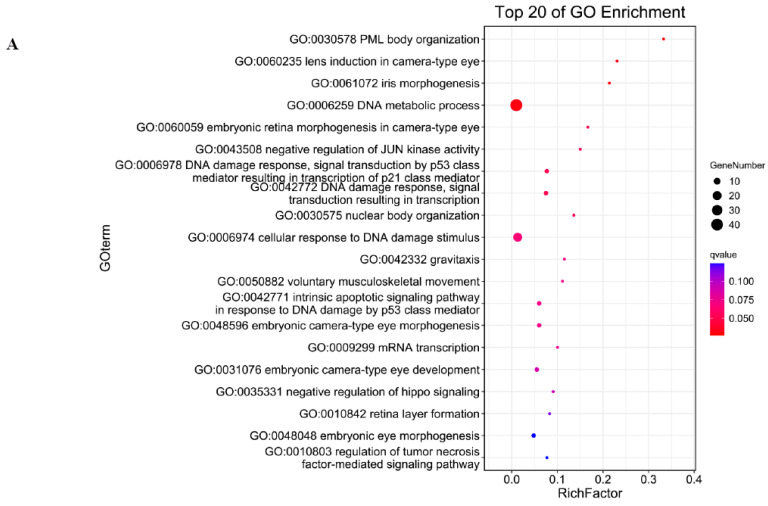

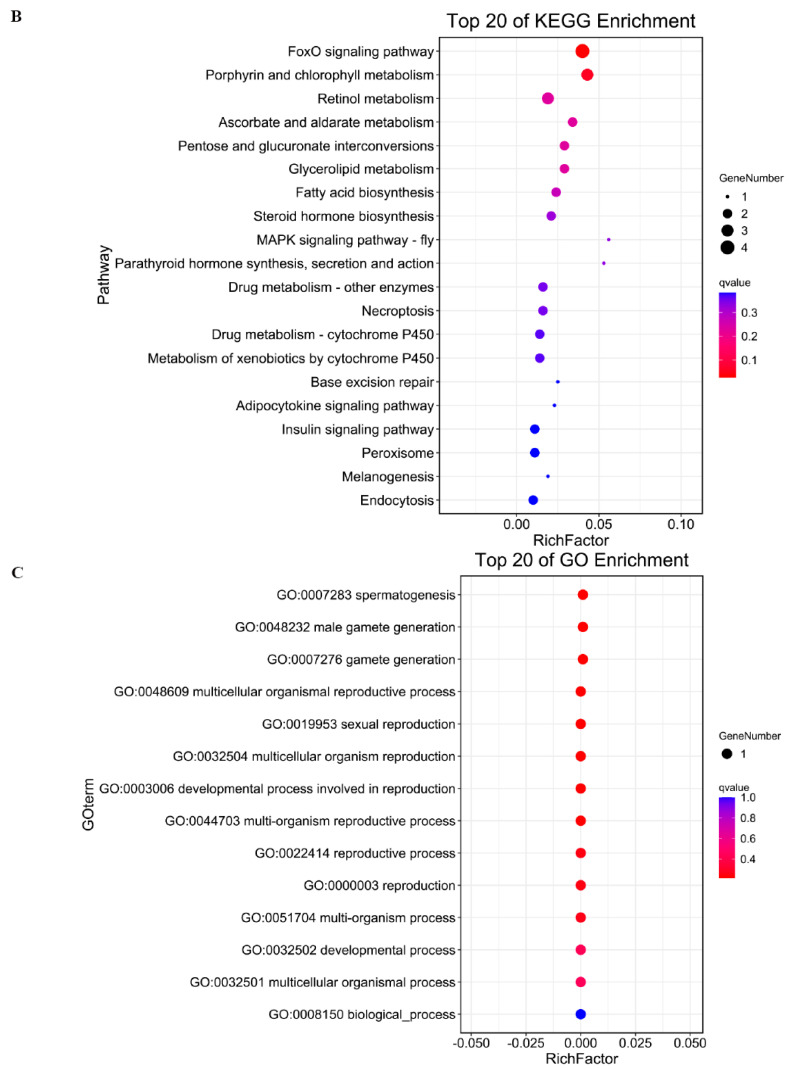
GO enrichment analysis of antisense target genes (**A**); KEGG analysis of antisense target genes (**B**); and GO analysis of antisense target genes in DEL-DEG pairs (**C**).

To reveal the interaction between antisense lncRNA and mRNA, we predicted the complementary binding. Predicting the antisense effects of all lncRNAs, a total of 356 lncRNA–mRNA pairs were obtained. Among them, 309 lncRNAs may have antisense effects on 294 mRNAs (Table S6). Biological process of GO (Gene Ontology) analysis showed the target genes of antisense lncRNAs were most enriched in PML body organization, DNA metabolic process and embryonic development (Figure 3A). KEGG (Kyoto Encyclopedia of Genes and Genomes) analysis showed the targeted genes could regulate FoxO signaling pathway, Porphyrin and chlorophyII metabolism, Retinol metabolism (Figure 3B). We predicted the antisense effects of differentially expressed lncRNAs and obtained two DEL–DEG pairs (Table S7). GO analysis showed the targeted genes enriched in spermatogenesis, male gamate generation and gamete generation (Figure 3C).

**Figure 4:**
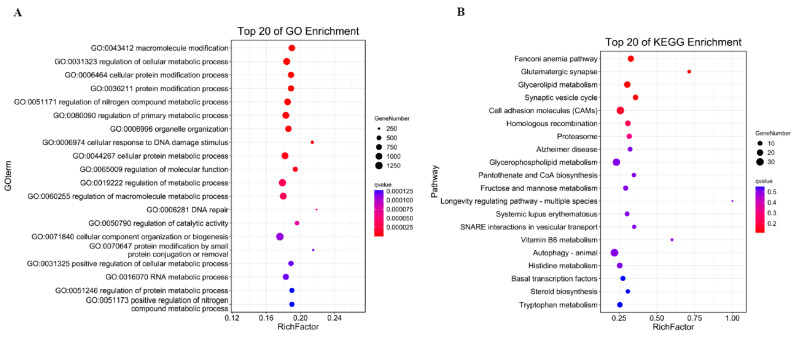

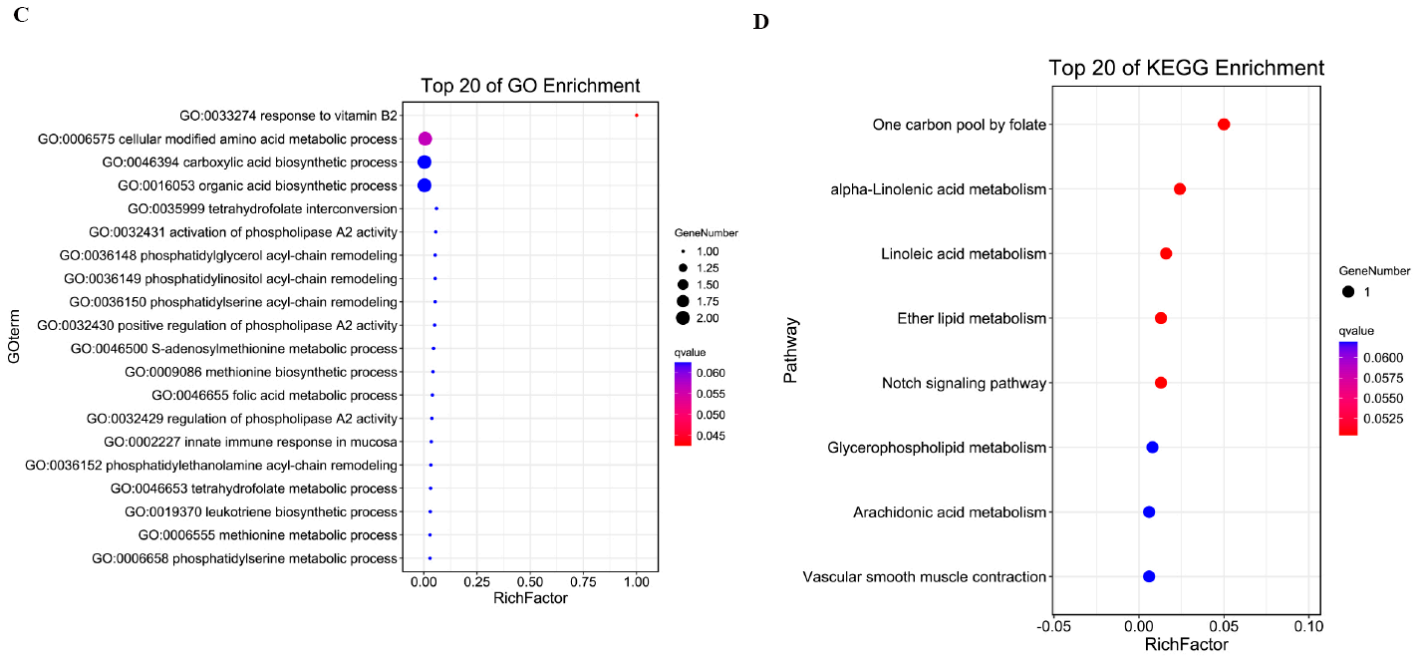
GO enrichment analysis of cis-activity target genes (**A**); KEGG analysis of cis-activity target genes (**B**); GO analysis of cis-activity target genes in DEL–DEG pairs (**C**); and KEGG analysis of cis-activity target genes in DEL–DEG pairs (**D**).

The fundamental mechanism underlying cis-acting target gene prediction relies on the functional association between long non-coding RNAs (lncRNAs) and their adjacent protein-coding genes. The results of all lncRNAs’ cis-activity predictions showed that 6928 lncRNA–mRNA pairs were achieved. Among the pairs, 4664 lncRNAs may have antisense effects on 5277 mRNAs (Table S8). The targeted genes were functionally implicated in macromolecular modification (including post-translational protein alterations and nucleic acid editing), modulation of cellular metabolic pathways, as well as protein processing and remodeling within cellular systems, demonstrating their critical roles in fundamental biological processes (Figure 4A). KEGG analysis showed the target genes mostly enriched in Fanconi anemia pathway, Glutamatergic synapse, and Glycerolipid metabolism (Figure 4B). Through systematic examination of genomic loci within 10 kb flanking regions of differentially expressed lncRNAs (DELs), we identified co-localized differentially expressed genes (DEGs) as potential cis-regulatory targets, yielding nine significant DEL–DEG genomic associations. Among these, eight DELs exhibited characteristic cis-acting regulatory patterns toward their corresponding DEG partners (Table S9). Functional annotation demonstrated pronounced enrichment of these target genes in three interconnected metabolic hierarchies: cellular modified amino acid metabolism, carboxylic acid biosynthesis, and organic acid biosynthetic pathways (Figure 4C). KEGG pathway mapping further established their involvement in folate-mediated one-carbon metabolism, α-linolenic acid metabolic regulation, and ether lipid metabolism networks (Figure 4D), suggesting coordinated regulation of these biochemical cascades by lncRNA-mediated cis-regulatory mechanisms.

**Figure 5:**
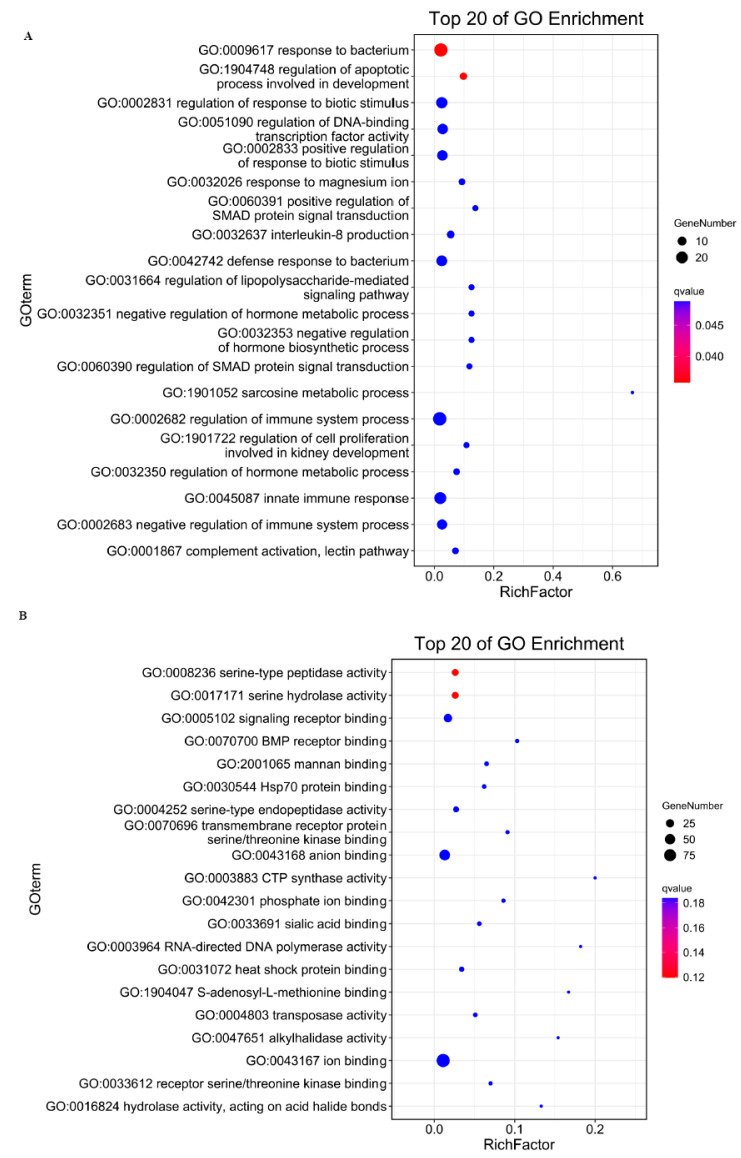
GO enrichment analysis of trans-target gene under biological process classification (**A**) and molecular function classification (**B**).

The trans-regulatory paradigm for lncRNA target prediction is predicated on functional correlations between lncRNAs and co-expressed protein-coding genes. Systematic correlation analysis of differentially expressed lncRNAs (DELs) and mRNAs (DEGs) identified 6159 potential trans-regulatory pairs, with computational models predicting 212 DELs exhibiting trans-acting regulation over 316 target DEGs (Table S10). Gene Ontology enrichment revealed these targets are significantly implicated in three key biological processes: developmental apoptosis regulation (GO:0008625), modulation of DNA-binding transcription factor activity (GO:0051090), and control of cell proliferation during nephrogenesis (GO:0072218), as illustrated in Figure 5A. Molecular function prediction results showed the targeted genes mostly enriched in serine-type peptidase activity, signaling receptor binding and BMP receptor binding (Figure 5B).

### 3.4. Experimental Verification of Differentially Expressed lncRNAs and mRNAs via Quantitative Real-Time PCR

**Figure 6:**
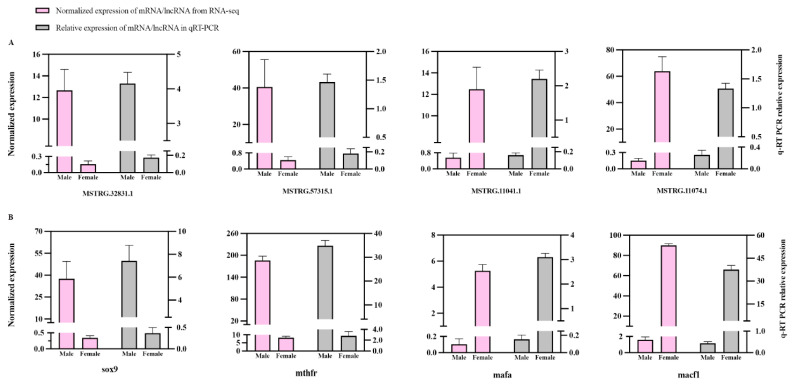
Comparison of expression levels for the four DEGs related to reproduction using qRT-PCR and RNA-seq (FPKM) (**A**); comparison of expression levels for four lncRNAs and their corresponding target gene in (**A**) using qRT-PCR and RNA-seq (FPKM) (**B**). All data were displayed as mean ± SE.

Among the aforementioned lncRNA–mRNA pairs (Tables S7, S9, and S10), four cis-regulatory DEL–DEG pairs implicated in reproductive development (MSTRG.32831.1-*sox9*, MSTRG.57315.1-*mthfr*, MSTRG.11041.1-*mafa*, MSTRG.11074.1-*macf1*) were subjected to qRT-PCR validation to confirm their sexually dimorphic expression profiles between testicular and ovarian tissues. Quantitative analysis demonstrated complete concordance between the experimentally validated expression patterns and RNA-seq-derived transcriptional profiles (Figure 6A,B). qPCR analysis showed a distinct expression pattern across eight transcripts: *sox9*, *mthfr*, MSTRG.32831.1, and MSTRG.57315.1 were highly expressed in testis, whereas *mafa*, *macf1*, MSTRG.11041.1, and MSTRG.11074.1 were highly expressed in ovary. Notably, these genes exhibiting gender-biased expression were all positively regulated by lncRNAs, which played a significant role in gonad development.

## 4. Discussion

Utilizing high-throughput sequencing, this study reports the lncRNA expression profile in sea cucumber gonads. From six constructed libraries, we obtained 420 million reads. These data supplement knowledge of the sea cucumber’s small RNA genome and provide a basis for investigating lncRNA regulation during gonadal development. We identified 212 lncRNAs significantly differentially expressed between testis and ovary tissues, which target thousands of genes involved in gonad development—an expected result considering the process’s complexity. Similarly to our results, it has been reported that Lnc133 may be involved in the gonadal development of pearl oyster *Pinctada martensii* [36]. LncPV13 played a role in the vitellogenesis of banana shrimp *Fenneropenaeus mergulensis* [37]. Whole transcriptome RNA sequencing provided 1502 lncRNAs during ovarian development in mud crab *Scylla paramamosain* [38]. Comparative transcriptome analysis provided insights into differentially expressed lncRNAs between ovary and testis of the mud crab [39]. LncRNAs functioned in cell differentiation during early development of Pacific oyster *Crassostrea gigas*, as well as sex differentiation and reproduction [40]. However, the role of lncRNAs in sex determination and gonad development within sea cucumber remained unclear [41].

lncRNAs were involved in regulating many post-transcriptional processes, which are related to complementary base pairing. Predictions identified target DEGs of DELs operating through antisense, cis- and trans-acting regulatory mechanisms. We identified 2 antisense, 8 cis and 212 trans DEL–DEG pairs in our analysis. Consistent with our research and methodology, lncRNA-target genes interplay regulated intestinal regeneration in the sea cucumber *A. japonicus.* A total of 2361 lncRNAs were identified, 183 of which were differentially expressed (DE-lncRNAs). The genes targeted by these lncRNAs were either cis- or trans-acting [42]. Transcriptome analysis revealed the lncRNA–mRNA co-expression network regulating the aestivation of sea cucumber. A specific trans-acting lncRNA (lncrna.1393.1) was identified as a potential regulator of Klf2 and Egr1 [12]. Strand-specific high-throughput sequencing was employed to analyze transcriptomic data from degenerated intestines of *A. japonicus* under starvation conditions. High-quality lncRNAs were identified and classified, and key differentially expressed mRNAs and lncRNAs associated with intestinal degradation were screened [43]. In our research, GO analysis showed two antisense targeted genes enriched in spermatogenesis, male gamate generation and gamete generation. qRT-PCR results of MSTRG.32831.1 and *sox9* confirmed with the above findings. Upstream lncRNAs may overlap with promoters or other cis-acting elements of co-expressed genes, thereby regulating gene expression at the transcriptional or post-transcriptional level. lncRNAs located in the 3′UTR or downstream of a gene may participate in other regulatory functions [44]. Therefore, we annotated lncRNAs, previously annotated, as located in the “unknown region.” If these lncRNAs are located within 10 kb upstream or downstream of a gene, they may overlap with regions containing cis-acting elements, thereby participating in the process of transcriptional regulation [45]. Eight DELs were predicted to have cis-acting effects on eight DEGs, which were enriched in cellular modified amino acid metabolic process. Transcriptome analysis showed that DEGs in gonads of the sea cucumber *A. japonicus* were mainly associated with amino acid metabolism [46]. It was consistent with the findings of research on sea cucumber *A. japonicus* [47] and *Holothuria scabra* [48]. MSTRG.57315.1-*mthfr* were identified as a cis-acting pair, which showed testis-biased expression pattern by qRT-PCR analysis. Trans-acting DEL–DEG pairs were mostly enriched in regulation of apoptotic process involved in development. The trans-acting pair MSTRG.11041.1-*mafa* and MSTRG.11074.1-*macf1* showed ovary-preferential expression, as validated by qRT-PCR analysis. lncRNAs were shown to mediate *H. leucospilota* gender differentiation via antisense, trans and cis-regulatory mechanisms targeting specific genes. Classified within Echinodermata (the vertebrate-proximal invertebrate phylum), *H. leucospilota* enables critical evolutionary analysis. Our research further elucidated the conservation of sophisticated lncRNA regulatory mechanisms governing reproduction throughout invertebrate–vertebrate evolution.

Quantitative real-time polymerase chain reaction (qRT-PCR) validation confirmed the transcriptional profiles of four differentially expressed lncRNA-target gene pairs. These molecular pairs exhibited significant expression differences, with functional annotations indicating their critical involvement in gonadal development processes. The target genes included *sox9*, *mthfr*, *mafa*, and *macf1* participated in gonadal development. Consistent with our findings, it has been reported that *sox9* expression gradually increased with testis development in sea cucumber *A. japonicus* [49]. Expression of *sox9* during Gut Regeneration in sea cucumber holothurian *Eupentacta fraudatrix* showed *sox9* may be involved in the regulation of the initial stages of transdifferentiation [50]. The enzyme methylenetetrahydrofolate reductase (MTHFR), crucial for folate metabolism, synthesizes 5-methyltetrahydrofolate—folate’s primary form in circulation. This molecule is necessary both for keeping homocysteine at safe levels and for furnishing one-carbon units essential for methylation. In mammals, mice lacking *mthfr* impaired testicular development [51]. Among aquatic animals, *mthfr* was essential for zebrafish embryonic development [52]. The function of *mthfr* in sea cucumbers has not yet been reported. Our study illustrated that *mthfr* influenced testis development of *H. leucospilota.* V-maf avian musculoaponeurotic fibrosarcoma oncogene homolog A (*mafa*) participated in the ovarian maturation of humans [53] and mice [54]. Currently, functional studies on *mafa* were rarely reported in sea cucumbers or even invertebrates. The Macrophin family, a giant cytoskeletal crosslinking protein and the second member of a novel subfamily within the plakin family, was encoded by *macf* gene [55]. The differential expression pattern of *macf1* in the maturation of mouse ovaries indicated that it was involved in the formation of oogonial cytoskeleton development [56].

The current understanding of lncRNA functions mainly relies on computational predictions, and more in-depth molecular biology experiments will be needed in the future to verify the specific regulatory mechanisms of lncRNA–mRNA pairs. In subsequent experiments, we could conduct dual luciferase reporter assays to validate the downstream regulatory factors of lncRNAs. Immunofluorescence experiments could help us localize lncRNA–mRNA pairs to specific sites within gonadal tissues.

## 5. Conclusions

In summary, this investigation systematically characterized the global lncRNA expression landscape during gonadal differentiation in the sea cucumber *H. leucospilota*. Transcriptomic analysis identified four significantly differentially expressed lncRNA–mRNA regulatory pairs exhibiting critical functional involvement in gonadgenesis. This study reported, for the first time, the differentially expressed lncRNA–mRNA pairs in the gonads of sea cucumber *H. leucospilota*. The differentially expressed lncRNA–mRNA in gonads of *H. leucospilota*, a representative tropical sea cucumber species, will reveal the molecular regulatory mechanisms underlying sex determination and gonadal development in sea cucumbers, providing crucial insights into the reproductive evolution of echinoderms.

## Data Availability

The datasets generated and analyzed in this study will be available in the NCBI SRA repository after 30 October 2025 (https://www.ncbi.nlm.nih.gov/bioproject/PRJNA1198409).

## Supplementary Materials

The following supporting information can be downloaded at: https://www.mdpi.com/article/10.3390/genes16111293/s1, Table S1. Primer sequences for reverse transcription experiment. Table S2. Primer sequences for qRT-PCR validation. Table S3. The statistics of lncRNA sequencing data. Table S4. Type of lncRNAs in the gonads of *H. leucospilota*. Table S5. The differential expressed lncRNAs between testis and ovary in *H. leucospilota*. Table S6. The differential expressed mRNAs between testis and ovary in *H. leucospilota*. Table S7. Antisense effect of lncRNA-mRNA pairs. Table S8. Antisense effect of DEL-DEG pairs. Table S9. Cis-acting effect of lncRNA-mRNA pairs. Table S10. Cis-acting effect of DEL-DEG pairs. Table S11. Trans-acting effects of DEL-DEG pairs.

## Abbreviations

The following abbreviations were used in this manuscript:

lncRNAs	Long Non-coding RNAs
DELs	Differentially Expressed lncRNAs
DEGs	Differentially Expressed Genes
PCA	Principal Component Analysis
GO	Gene Ontology
KEGG	Kyoto Encyclopedia of Genes and Genomes
FPKM	Fragments Per Kilobase of exon model per Million mapped fragments
